# A Monitoring Method to Evaluate the Accumulation of Antimicrobial‐Resistance Genes in Gram‐Negative Bacteria Distributed in Environmental Water

**DOI:** 10.1111/1758-2229.70265

**Published:** 2025-12-19

**Authors:** Nobuyoshi Yagi, Sora Miyazato, Nguyen Quoc Anh, Bui Thi Mai Huong, Itaru Hirai

**Affiliations:** ^1^ Laboratory of Clinical Physiology, School of Health Sciences, Faculty of Medicine University of the Ryukyus Okinawa Japan; ^2^ Laboratory of Microbiology, School of Health Sciences, Faculty of Medicine University of the Ryukyus Okinawa Japan; ^3^ Department of Food Microbiology and Molecular Biology National Institute of Nutrition Hanoi Vietnam

**Keywords:** antimicrobial‐resistance, antimicrobial‐resistant bacteria, environment, monitoring

## Abstract

Antimicrobial‐resistant bacteria (AMRB) distributed in the environment can cause human refractory infections. Sufficient monitoring of environmental AMRB had not been performed regularly, and a less burdensome and more efficient method for monitoring environmental AMRB was needed. Assuming to monitor AMRB in the environment, we selected 910 AMRB isolates using cefotaxime and ciprofloxacin from Vietnamese environmental water samples and subjected to 16S rRNA sequencing. It indicated that *Escherichia coli* (36.0%), *Citrobacter freundii* (21.4%), *Acinetobacter baumannii* (20.6%), and *Klebsiella pneumoniae* (19.7%) were dominant. Using *E. coli* as a model, we further analysed AMRB isolates by phylogenetic analysis and whole‐genome sequencing (WGS). The sequenced full‐length *fimH* of the isolates were plotted with the already published *fimH* sequences on a phylogenetic tree. Considering phylogeny, 14 *E. coli* strains were subjected to WGS that indicated not only the number and type of ARGs but also the order of ARGs on the plasmid were confirmed in the analysed *E. coli* isolates. More importantly, 3 of the 14 strains were *bla*_NDM‐5_‐positive that is, carbapenem‐resistant *E. coli* . These results suggest that our analytical procedure in this study is applicable as a monitoring method to understand in detail genetic characteristics of AMRB isolates in environmental water samples.

## Graphical abstract

There is less monitoring for antimicrobial‐resistance bacteria (AMRB) spread in the environment, probably due to costs. This study proposes a cost‐effective monitoring method based on isolating AMRB strains. This method enables us to understand the nature of environmental AMRB strains and assess their risk to humans and animals.

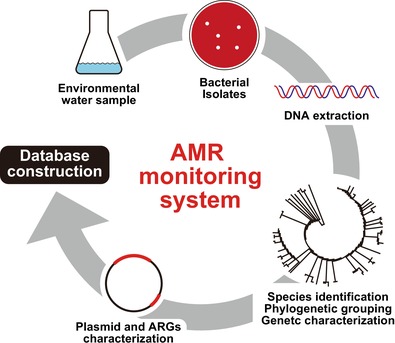

## 1 Introduction

As in case that extended‐spectrum β‐lactamase (ESBL)‐producing Enterobacteriaceae have been detected worldwide (Doi et al. 2017), it is supposed that certain types of AMRB are circulating among healthcare‐associated facilities, communities, and the environment (Ajose et al. 2024). The situation regarding AMRB could be worse, previous studies have reported that even carbapenem‐resistant and colistin‐resistant bacteria, which are resistant to the last resort drugs, carbapenems and colistin, respectively, were detected in the community (Biswas et al. 2023; Rhouma et al. 2023; Jean et al. 2022; Hansen 2021; Cherak et al. 2021; Yamamoto et al. 2019; Yamamoto et al. 2020). For these reasons, it is considered important to monitor AMRB in the environment as well as AMRB surveillance in healthcare‐associated facilities. In many regions and countries, surveillance of AMRB is mainly conducted in healthcare‐associated facilities, and the target AMRB species have been selected by considering whether bacterial species could cause health hazards to humans.

It seems that there is less report that certain countries and regions maintain actively monitoring system for AMRB spreading in the environment, food or healthy individuals. This is likely due to a lack of analytical systems to monitor AMRB at a reasonable cost, implying that fundamental budget to maintain the AMRB monitoring system might not be allocated.

We have previously conducted studies of AMRB, especially ESBL‐producing *E. coli* , in Ba Vi district, Hanoi city in Vietnam (Bui et al. 2015; Ueda et al. 2015) and reported that more than half of asymptomatic healthy Vietnamese living in the communities carried ESBL‐producing *E. coli* (Bui et al. 2015). Ba Vi district, a rural area of Hanoi city, is an area that maintains farming of the traditional VAC ecosystem, with each farmer's house having an attached livestock shed, farming area and a small pond (Hop le 2003). Because VAC ecosystem is a recycling‐based agriculture, it is important to know what kinds of AMRB are distributed in the ponds attached to the farmer's house. However, there is no AMRB monitoring at Vietnamese VAC ecosystem and it has been unclear what kinds of AMRB would be distributed in the ponds attached to VAC ecosystem.

Many studies have observed that various ARGs accumulated on plasmids that can be transferred beyond barriers among bacterial species. In addition, it is regarded that the acquisition of plasmids carrying these multiple ARGs by antimicrobial‐sensitive bacteria is one of the factors in the emergence of multidrug resistant bacteria (MRB) (Wang et al. 2024). Therefore, it is essential to understand which and how many ARGs would be accumulated on plasmid and to analyse the genetic background of AMRB to study the distribution mechanism of AMRB. Regarding the AMRB spread in the environment, in addition to isolation and analysis of AMRB by conventional microbiological methods, detecting ARGs in environmental water samples, such as river water or pond water have been increasingly performed. To detect ARGs in the environment, PCR is being replaced by next generation sequencing. Presently, next‐generation sequencing has been used in amplicon sequencing, environmental metabarcoding, metagenomics and so forth (Li and Yan 2023). These techniques are powerful enough to detect most of the existing ARGs and determine the bacterial species contained in each sample tested. However, environmental water, especially pond water, could sometimes be contaminated with several clones of several AMRB species, and multiple bacterial species may harbour certain plasmids belonging to the same incompatibility (Inc.) groups (Akiba et al. 2016). In such cases, even if metagenome‐assembled genomes would be successfully constructed from the obtained metagenome data, it could mislead investigators to identify which bacterial species harboured which plasmids.

Meanwhile, in our unpublished work, we established a WGS protocol, Shallow‐sequencing, for AMRB and it suggested that could sufficiently reduce the cost per strain (Yagi, Miyagi, et al. 2024). The established protocol allows us to analyse a greater number of AMRB and to evaluate how and how many ARGs have been accumulated on the antimicrobial‐resistance plasmids carried by AMRB. In this study, we proposed a method that could allow us to consider accumulation of ARGs in each AMRB isolate obtained from environmental water samples and discussed whether our proposed methods were applicable to AMRB monitoring.

## 2 Experimental Procedures

### 2.1 Structure of the Method

This study proposed a method that could evaluate ARG accumulation in Gram‐negative AMRB isolates obtained from environmental water. In this study, we selected *E. coli* as a model bacterial species to confirm the practicality of the proposed method that could evaluate ARG accumulation in Gram‐negative bacteria isolated from environmental water. The method consisted of sampling and enrichment culture, selection by antibiotics‐containing solid media, bacterial species identification for AMRB by 16S rRNA sequencing, phylogenetic grouping of AMRB by *fimH* sequencing, and whole genome sequencing with the following data analysis of the representative *E. coli* isolates.

### 2.2 Environmental Water Samples and Isolation of AMRB

Environmental water samples were collected from ponds in the BaVi area, Hanoi city, Vietnam. Basically, 50 mL of surface water was collected from each of 12 different ponds that were located at the farmer's houses maintaining the traditional VAC ecosystem in November 2023. The collected water samples were brought to the National Institute of Nutrition, Vietnam. A sterile cotton swab of a seed swab kit was dipped in each of the 12 collected environmental water samples and used for spreading onto one antibiotic‐free MacConkey agar plate prepared in a 90‐mm diameter Petri dish. After 24 h of incubation at 37°C, all the grown bacterial colonies on each Petri dish were resuspend in 2 mL of trypticase soy broth (TSB) containing 10% glycerol and stored at −80°C. One loop of the stocked bacteria was taken using disposable inoculating loop and diluted with TSB from 10^−1^ to 10^−5^. Each 100 μL of the diluted bacterial solution per one 90‐mm diameter Petri dish was used for inoculation on MacConkey agar containing 2 μg/mL of cefotaxime or 2 μg/mL of ciprofloxacin. The grown bacterial colonies that is, AMRB strains, were transferred into 96 well plates containing 150 μL of TSB containing 10% glycerol and stored at −20°C until analysis.

### 2.3 Amplification of Full‐Length of 16S rRNA Gene for Identification of Bacterial Species

To identify bacterial species of the collected AMRB strains, full‐length of 16S rRNA gene was amplified by gene specific primers (Table 1). Bacterial DNA was extracted by the conventional boiling method. Briefly, 1 mL of bacterial culture was centrifuged at 14,500 rpm for 1 min and the precipitated bacterial cells were resuspended in 0.5 mL of 10 mM Tris, HCl, pH 8.0, 1 mM EDTA buffer. After 5 min of incubation at 100°C, the bacterial suspension was centrifuged at 14,500 rpm for 10 min. Then, absorbance at 260 nm (A260) of the consequent supernatants were measured. Even though it would contain some contaminants, we assumed that a solution with A260 = 1.0 had a DNA concentration of 50 ng/mL, and the supernatants were diluted and adjusted at 1.0 ng/μL to prepare template DNA for PCR.

**TABLE 1:** Sequences of primers in this study.

Primer name	Sequence (5′–3′)
Forward Primer for FL‐16S rRNA	AGRGTTYGATYMTGGCTCAG
16S rRNA‐R‐2‐25	GGGCGATTTAGGTGACACTATAGCGTAAGTTGGGTATGCAACGCAATGCGGYTACCTTGTTACGACTT
16S rRNA‐R‐2‐26	GGGCGATTTAGGTGACACTATAGCCATACAGCGACTACGCATTCTCATCGGYTACCTTGTTACGACTT
16S rRNA‐R‐2‐27	GGGCGATTTAGGTGACACTATAGCCGACGGTTAGATTCACCTCTTACACGGYTACCTTGTTACGACTT
16S rRNA‐R‐2‐28	GGGCGATTTAGGTGACACTATAGCTGAAACCTAAGAAGGCACCGTATCCGGYTACCTTGTTACGACTT
16S rRNA‐R‐2‐29	GGGCGATTTAGGTGACACTATAGCCTAGACACCTTGGGTTGACAGACCCGGYTACCTTGTTACGACTT
16S rRNA‐R‐2‐30	GGGCGATTTAGGTGACACTATAGCTCAGTGAGGATCTACTTCGACCCACGGYTACCTTGTTACGACTT
16S rRNA‐R‐2‐31	GGGCGATTTAGGTGACACTATAGCTGCGTACAGCAATCAGTTACATTGCGGYTACCTTGTTACGACTT
16S rRNA‐R‐2‐32	GGGCGATTTAGGTGACACTATAGCCCAGTAGAAGTCCGACAACGTCATCGGYTACCTTGTTACGACTT
16S rRNA‐R‐2‐33	GGGCGATTTAGGTGACACTATAGCCAGACTTGGTACGGTTGGGTAACTCGGYTACCTTGTTACGACTT
16S rRNA‐R‐2‐34	GGGCGATTTAGGTGACACTATAGCGGACGAAGAACTCAAGTCAAAGGCCGGYTACCTTGTTACGACTT
16S rRNA‐R‐2‐35	GGGCGATTTAGGTGACACTATAGCCTACTTACGAAGCTGAGGGACTGCCGGYTACCTTGTTACGACTT
16S rRNA‐R‐2‐36	GGGCGATTTAGGTGACACTATAGCATGTCCCAGTTAGAGGAGGAAACACGGYTACCTTGTTACGACTT
16S rRNA‐R‐2‐37	GGGCGATTTAGGTGACACTATAGCGCTTGCGATTGATGCTTAGTATCACGGYTACCTTGTTACGACTT
16S rRNA‐R‐2‐38	GGGCGATTTAGGTGACACTATAGCACCACAGGAGGACGATACAGAGAACGGYTACCTTGTTACGACTT
16S rRNA‐R‐2‐39	GGGCGATTTAGGTGACACTATAGCCCACAGTGTCAACTAGAGCCTCTCCGGYTACCTTGTTACGACTT
16S rRNA‐R‐2‐40	GGGCGATTTAGGTGACACTATAGCTAGTTTGGATGACCAAGGATAGCCCGGYTACCTTGTTACGACTT
EC fimH‐F	ACGCCAATAATCGATTGCAC
EC fimH‐R‐25	GGGCGATTTAGGTGACACTATAGCGTAAGTTGGGTATGCAACGCAATGCGAGTTATTACCCTGTTTGCTG
EC fimH‐R‐26	GGGCGATTTAGGTGACACTATAGCCATACAGCGACTACGCATTCTCATCGAGTTATTACCCTGTTTGCTG
EC fimH‐R‐27	GGGCGATTTAGGTGACACTATAGCCGACGGTTAGATTCACCTCTTACACGAGTTATTACCCTGTTTGCTG
EC fimH‐R‐28	GGGCGATTTAGGTGACACTATAGCTGAAACCTAAGAAGGCACCGTATCCGAGTTATTACCCTGTTTGCTG
EC fimH‐R‐29	GGGCGATTTAGGTGACACTATAGCCTAGACACCTTGGGTTGACAGACCCGAGTTATTACCCTGTTTGCTG
EC fimH‐R‐30	GGGCGATTTAGGTGACACTATAGCTCAGTGAGGATCTACTTCGACCCACGAGTTATTACCCTGTTTGCTG
EC fimH‐R2‐31	GGGCGATTTAGGTGACACTATAGCTGCGTACAGCAATCAGTTACATTGCGAGTTATTACCCTGTTTGCTG
EC fimH‐R‐32	GGGCGATTTAGGTGACACTATAGCCCAGTAGAAGTCCGACAACGTCATCGAGTTATTACCCTGTTTGCTG
EC fimH‐R‐33	GGGCGATTTAGGTGACACTATAGCCAGACTTGGTACGGTTGGGTAACTCGAGTTATTACCCTGTTTGCTG
EC fimH‐R‐34	GGGCGATTTAGGTGACACTATAGCGGACGAAGAACTCAAGTCAAAGGCCGAGTTATTACCCTGTTTGCTG
EC fimH‐R‐35	GGGCGATTTAGGTGACACTATAGCCTACTTACGAAGCTGAGGGACTGCCGAGTTATTACCCTGTTTGCTG
EC fimH‐R‐36	GGGCGATTTAGGTGACACTATAGCATGTCCCAGTTAGAGGAGGAAACACGAGTTATTACCCTGTTTGCTG
EC fimH‐R‐37	GGGCGATTTAGGTGACACTATAGCGCTTGCGATTGATGCTTAGTATCACGAGTTATTACCCTGTTTGCTG
EC fimH‐R‐38	GGGCGATTTAGGTGACACTATAGCACCACAGGAGGACGATACAGAGAACGAGTTATTACCCTGTTTGCTG
EC fimH‐R‐39	GGGCGATTTAGGTGACACTATAGCCCACAGTGTCAACTAGAGCCTCTCCGAGTTATTACCCTGTTTGCTG

To amplify full‐length of 16S rRNA, GoTaq Green Master Mix (Promega K.K., Tokyo, Japan) was used by following the manufacture's instruction. Briefly, 1 ng of the extracted bacterial DNA was used as the template in 10 μL of reaction mixture containing forward primer and one of the barcoded‐reverse primers (Table 1). Thermal conditions for amplifying full‐length 16S rRNA was as follows, an initial melt of 98°C for 2 min, 30 cycles of 98°C for 10 s, 55°C for 30 s and 72°C for 1 min 45 s, and a final extension at 72°C for 7 min. Then the amplified DNA samples were purified by KAPA Pure Beads (Roche Sequencing Solutions Inc., Pleasanton, CA) and subjected to the Nanopore sequencing (Oxford Nanopore Technologies, Oxford, UK).

### 2.4 Amplification of *E. coli* by *fimH* for Phylogenetic Classification

To amplify *fimH*, 1 ng of the extracted bacterial DNA was used as the template in 10 μL of GoTaq Green Master Mix reaction mixture containing forward primer and one of the barcoded‐reverse primers (Table 1), and performed thermal cycles consist of an initial melt of 98°C for 2 min, 30 cycles of 98°C for 10 s, 53°C for 30 s and 72°C for 1 min, and a final extension at 72°C for 7 min. The amplified DNA samples were purified by KAPA Pure Beads by following the manufacture's instruction and subjected to the Nanopore sequencing.

### 2.5 Bacterial Genomic DNA Purification

Bacterial genomic DNA of the representative AMRB strains were purified by the Monarch Spin gDNA Extraction Kit (New England Biolabs., Ipswich, MA) by following the manufacture's instruction.

### 2.6 Library Preparation and Nanopore Sequencing

The purified amplified DNA or bacterial genomic DNA were subjected to Nanopore sequencing. For Nanopore sequencing 65 ng of the amplified DNA or 200 ng of the bacterial genomic DNA was used to prepare libraries by following the protocol of the Native Barcoding Kit 24 V14 provided by Oxford Nanopore Technologies. Nanopore sequencing was performed using the Flongle Flow Cell (R10.4.1).

### 2.7 Sequence Read Clean‐up

The obtained sequence reads were demultiplexed based on barcode sequences using Porechop (Wick et al. 2017) and the demultiplexed that is, de‐barcoded sequence reads were used for following analysis. The de‐barcoded sequence reads were subjected to quality filtering with quality scores and read length. For 16S rRNA gene sequencing and WGS of AMRB strains, sequence reads with a quality score of at least 10 and a read length of 1.0 k bp or more were used. For *fimH* typing, only sequence reads with a quality score of at least 14 and a read length of 1.2 k bp or more were used in the analysis.

### 2.8 Bacterial Species Identification Using 16S rRNA Sequences

Bacterial species identification based on the 16S rRNA gene sequence was performed using Emu (Curry et al. 2022). Among the emu analysis results, only data with an “abundance” of 95% or higher and “estimated counts” of 100 or higher were selected.

### 2.9 *fimH* Typing

The cleaned sequence reads were analysed using Amplicon_Sorter (Vierstraete and Braeckman 2022) to obtain consensus *fimH* sequences of *E. coli* isolates obtained in this study. These consensus sequences were then analysed with FimTyper (Roer et al. 2017) to determine the fimH type. All *fimH* sequences that were registered in the FimTyper database were clustered at a cut off value (99% identity). A phylogenetic tree was constructed using the clustered *fimH* sequences and the *fimH* sequences of the determined fimH types. MEGA11 was used for phylogenetic tree construction (Tamura et al. 2021).

### 2.10 WGS of AMRB Isolates and Data Analysis

De novo assembly was performed using Flye (Kolmogorov et al. 2019) and the cleaned sequence reads originating from the representative AMRB strains. The obtained draft assemblies were polished using medaka (https://github.com/nanoporetech/medaka) to generate consensus sequences that is, contigs. The generated contigs of the representative AMRB strains were subjected to identifying chromosome‐derived open reading frames (ORFs) (Yagi, Uechi, and Hirai 2024).

The ORFs of each representative AMRB strain were detected by blastn (Altschul et al. 1990) and a ORF database included all ORFs that was constructed with PanTA (Le et al. 2023) and 5000 complete *E. coli* genome sequences retrieved from NCBI datasets. The detected and absent (not detected) ORFs were assigned a value of 1 and 0, respectively, collected in an ORF presence‐absence table, and were converted to binary sequences for each *E. coli* genome sequence. The binary sequences were analysed using the BINNGAMMA model in RAxML (Stamatakis 2014). The resulting data were visualised using R and RStudio to construct an ORF tree.

ARGs and plasmid replicons were detected using AMRFinderPlus (Feldgarden et al. 2021) and PlasmidFinder (Carattoli et al. 2014), respectively. The contigs, in which ARGs were detected, were assigned as plasmid‐derived contigs using PLASMe (Tang et al. 2023).

Plasmid sequences were retrieved from the RefSeq database by keyword search using “complete sequence,” filtered by species: “bacteria”, genetic compartment: “plasmid”, and sequence length: 3000–500,000 bp. BLAST searches were performed to identify plasmid sequences homologous to the detected plasmid‐derived contigs. The plasmid sequence with the highest % identity and query cover to each plasmid‐derived contig was designated as the reference plasmid sequence. The similarity among the plasmid sequences, the reference sequences, and the plasmid‐derived contigs were assessed using BRIG (Alikhan et al. 2011). In addition, the ARG cluster between the plasmid reference sequences and the plasmid‐derived contigs was drawn using Clinker (Gilchrist and Chooi 2021), based on GenBank files annotated with the BacAnt (Hua et al. 2021).

## 3 Results

In this study, we isolated 910 bacterial colonies from the collected 12 environmental water samples and subjected to 16S rRNA amplification to identify bacterial species. Consequently, bacterial species of 791 (86.9%) of the 910 isolates were identified. *E. coli* and *K. pneumoniae* were detected both in the CTX‐resistant and CIP‐resistant isolates. Most of *C. freundii* and *A. baumannii* were mainly detected in CIP‐resistant and CTX‐resistant isolates, respectively (Table 2).

**TABLE 2:** Identified bacterial species of the isolates obtained from the ponds and rivers.

Identified isolates	Total		CTX‐resistant isolates			CIP‐resistant isolates		
	*n*		*n*	%^a^		*n*	%^a^	
	791		378	47.8		413	52.2	
Bacterial spp.	*n*	%^a^	*n*	%^b^	%^c^	*n*	%^b^	%^c^
*Escherichia coli*	285	36.0	169	59.3	44.7	116	40.7	28.1
*Citrobacter freundii*	169	21.4	10	5.9	2.6	159	94.1	38.5
*Acinetobacter baumannii*	163	20.6	162	99.4	42.9	1	0.6	0.2
*Klebsiella pneumoniae*	156	19.7	23	14.7	6.1	133	85.3	32.2
*Acinetobacter. Pittii*	3	0.4	2	66.7	0.5	1	33.3	0.2
*Acinetobacter soli*	8	1.0	8	100.0	2.1	0	0.0	0.0
Others	7	0.9	4^d^	57.1	1.1	3^e^	42.9	0.7

^a^ Rations in the total number of the AMRBs.

^b^ Ratios in the total number of the identified species.

^c^ Ratios in the CTX‐ or CIP‐resistant isolates.

^d^ *Aeromonas caviae* .

^e^ *Enterobacter hormaechei* .

**FIGURE 1:**
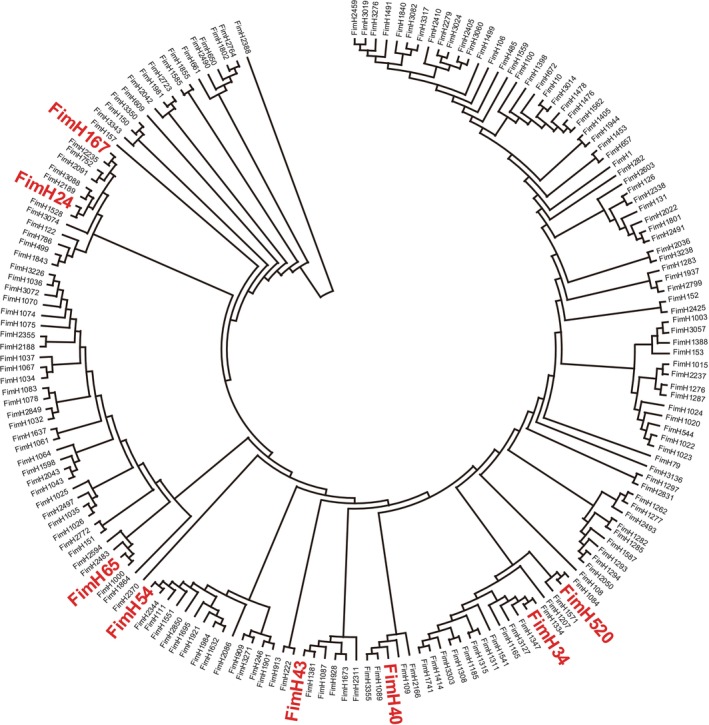
The *fimH* phylogenetic tree. The phylogenetic tree was constructed by the analysed fimH sequence of the 285 *E. coli* isolates and representative *fimH* sequences collected from the FimTyper database. The 185 *fimH* sequences were classified into eight fimH types indicated by red colour.

For the steps after species identification by 16S rRNA sequencing, the results for the model species, *E. coli* , were described. Firstly, we performed phylogenetic grouping by *fimH* of *E. coli* (Figure 1) to assess which genetic lineages the *E. coli* strains obtained in this study were closest to lineages of previously reported strains. As shown in Figure 1 and Table S1, a total of 285 *E. coli* isolates were classified into eight *fimH* types. The numbers of strains contained in each *fimH* type, fimH24, fimH34, fimH40, fimH43, fimH54, fimH65, fimH167, and fimH520, were 86 (30.2%), 50 (17.5%), 14 (4.9%), 6 (2.1%), 25 (8.8%), 65 (22.8%), 8 (2.1%) and 1 (0.4%), respectively. For the remaining 32 (11.2%) *E. coli* isolates, the *fimH* type was not determined. We confirmed relative locations of the eight fimH types observed in this study on a phylogenetic tree constructed using sequence data obtained from the public database, Genbank (Figure 1). It was found that these eight fimH types appeared to be positioned separately from each other on the phylogenetic tree. Therefore, in this study, we selected a total of 14 *E. coli* isolates by considering *fimH* type distribution and subjected to WGS.

**FIGURE 2:**
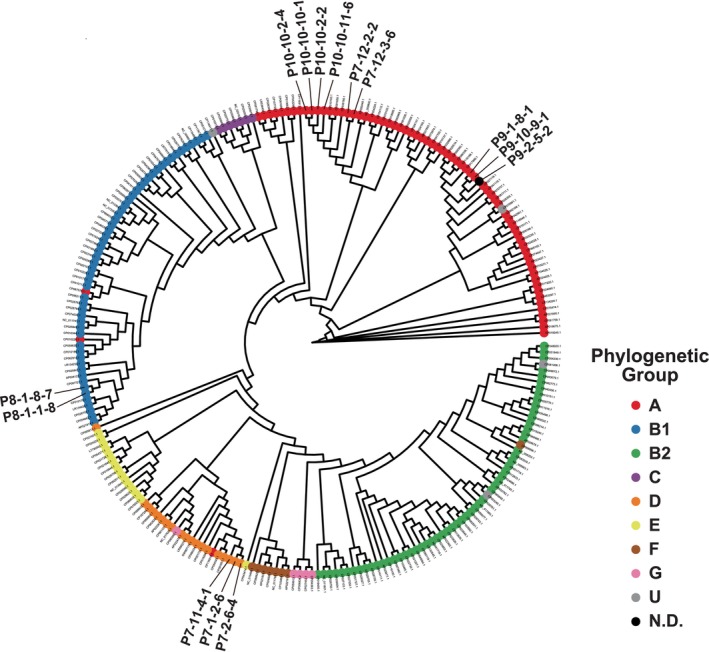
Comparison of genetic characteristics of *E. coli* isolates by an ORF tree. Genetic characteristics of the 14 analysed *E. coli* isolates were compared with those of the 227 *E. coli* reference genome sequences collected from RefSeq by an ORF tree. For drawing ORF trees, an ORF database was constructed with PanTa and 5000 complete *E. coli* genome sequences retrieved from NCBI datasets. The present and absent ORFs in each *E. coli* isolate genome was assigned a value of 1 and 0, respectively. Then it was converted to a binary sequence for each *E. coli* genome sequence. The binary sequences were analysed using the BINNGAMMA model in RAxML and an ORF tree was drawn by R and RStudio.

The genetic information of the 14 selected strains was collected by WGS. ORFs were extracted from the genetic information of the 14 analysed strains. Binary sequences were obtained based on the presence or absence of ORF from the genetic information of the analysed strains, and a phylogenetic tree was depicted combined with binary sequences obtained from the already published sequences with their phylogenetic groups (Figure 2 and Table S2). Judging from the phylogenetic tree depicted by the ORFs, it seemed that the 14 selected *E. coli* isolates were classified into phylogenetic group A (nine isolates, 64.3%), B1 (two isolates, 14.3%), and D (three isolates, 21.4%).

**FIGURE 3:**
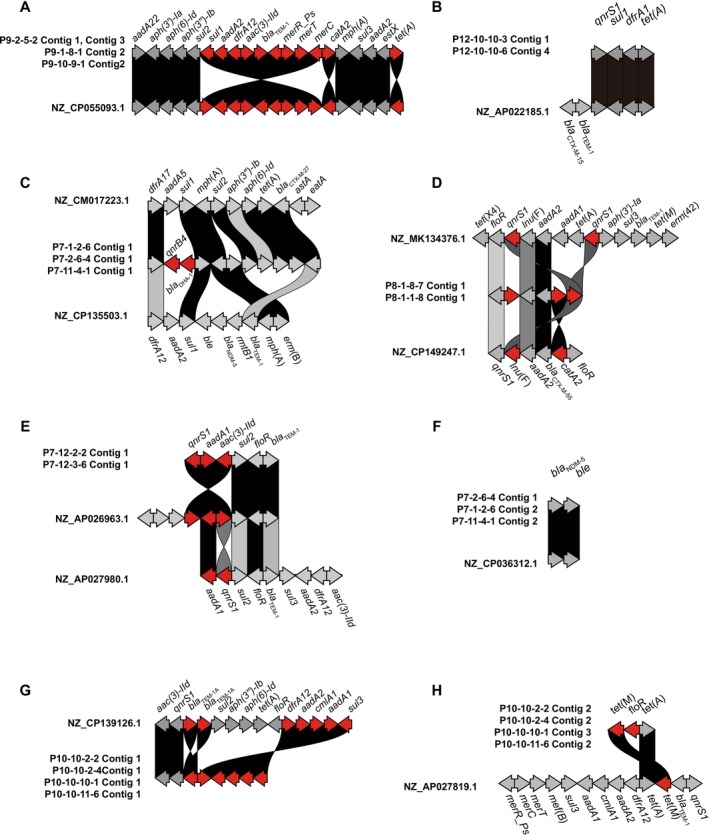
Comparison of gene clusters detected in the whole genome information of the 14 analysed *E. coli* strains. The gene cluster plots were drawn by clinker to compare the order of ARGs, To simplify comparison of the ARGs arrangement, the gene scale was not considered. Red arrow means the difference between plasmid‐derived contigs and reference sequences. (A) P9‐2‐5‐2 Contig 1and Contig 3, P9‐1‐8‐1 Contig 2, P9‐10‐9‐1 Contig2, (B) P12‐10‐10‐3 Contig 1, P12‐10‐10‐6 Contig 4, (C) P7‐1‐2‐6 Contig 1, P7‐2‐6‐4 Contig 1, P7‐11‐4‐1 Contig 1, (D) P8‐1‐8‐7 Contig 1, P8‐1‐1‐8 Contig 1, (E) P7‐12‐2‐2 Contig 1, P7‐12‐3‐6 Contig 1, (F) P7‐2‐6‐4 Contig 1, P7‐1‐2‐6 Contig 2, P7‐11‐4‐1 Contig 2, (G) P10‐10‐2‐2 Contig 1, P10‐10‐2‐4Contig 1, P10‐10‐10‐1 Contig 1, P10‐10‐11‐6 Contig 1, (H) P10‐10‐2‐2 Contig 2, P10‐10‐2‐4 Contig 2, P10‐10‐10‐1 Contig 3, P10‐10‐11‐6 Contig 2.

In addition to the genetic lineages of the 14 *E. coli* isolates analysed, the WGS provided information related to ARGs and plasmid replicons (Table 3). In this study, any constructed contig that contained at least one ARG that is, AMR Contig, was subjected to BLAST search to clarify whether the contig belonged to chromosome or plasmid. As shown in Table 3, 34 contigs were constructed from the 14 *E. coli* isolates sequenced. BLAST search indicated 8 (23.5%) and 26 (76.5%) of the 34 contigs were classified into chromosome and plasmid, respectively. It was suggested that each of the analysed *E. coli* isolates possessed one to three plasmids, and that each plasmid carried 2–13 ARGs. Three *E. coli* isolates (P7‐1‐2‐6, P7‐2‐6‐4, P7‐11‐4‐1) possessed *bla*_CTX‐M_s and *bla*_DHA‐1_, in addition to *bla*_NDM‐5_ (Table 3, Figure 3). The WGS indicated gene clusters in which the detected ARGs were arranged on the plasmids in the order shown in Table 3.

**TABLE 3:** Detected ARGs and their order on the constructed contigs.

Isolate ID	AMR contig ID	Numver of the detected ARGs	Order of the detected ARGs in each contig	Detected plasmid replicons	Chromosome or plasmid	The reference plasmid sequence^a^
P7‐1‐2‐6	1	11	*dfrA17*, *qnrB4*, *bla*_DHA‐1_, *sul1*, *mph*(A), *erm*(B), *sul2, aph(3″)‐Ib*, *aph(6)‐Id*, *tet*(A), *bla*_CTX‐M‐27_	IncFIB(pB171), IncFII(pRSB107)	Plasmid	NZ_CP135503.1
	2	2	*bla*_NDM‐5_, *ble*	IncX3, Col156	Plasmid	NZ_CP036312.1
P7‐2‐6‐4	1	11	*dfrA17*, *qnrB4*, *bla*_DHA‐1_, *sul1*, *mph*(A), *erm*(B), *sul2*, *aph(3″)‐Ib*, *aph(6)‐Id*, *tet*(A), *bla*_CTX‐M‐27_	IncFIA, IncFIB(pB171), IncFII(pRSB107)	Plasmid	NZ_CP135503.1
	2	2	*bla*_NDM‐5_, *ble*	IncX3	Plasmid	NZ_CP036312.1
P7‐11‐4‐1	1	11	*dfrA17*, *qnrB4*, *bla*_DHA‐1_, *sul1*, *mph*(A), *erm*(B), *sul2*, *aph(3″)‐Ib*, *aph(6)‐Id*, *tet*(A), *bla*_CTX‐M‐27_	IncFIA, IncFIB(pB171), IncFII(pRSB107)	Plasmid	NZ_CP135503.1
	2	2	*bla*_NDM‐5_, *ble*	IncX3	Plasmid	NZ_CP036312.1
P7‐12‐2‐2	1	6	*qnrS1*, *aadA1*, *aac(3)‐IId*, *sul2*, *floR*, *bla*_TEM‐1_	IncR, IncX1	Plasmid	NZ_AP027980.1
P7‐12‐3‐6	1	6	*qnrS1*, *aadA1*, *aac(3)‐IId*, *sul2*, *floR*, *bla*_TEM‐1_	IncR, IncX1	Plasmid	NZ_AP026963.1
P8‐1‐1‐8	1	6	*bla*_CTX‐M‐55_, *qnrS13*, *floR*, *aac(3)‐IId*, *lnu*(F), *aadA2*	IncFIB(AP001918), IncFIC(FII)	Plasmid	NZ_CP149247.1
P8‐1‐8‐7	1	5	*qnrS*, *bla*_CTX‐M_, *aadA2*, *aac(3)‐IId*, *floR*	IncFIB(AP001918), IncFIC(FII)	Plasmid	NZ_CP149247.1
	2	2	*bla*_LAP‐2_, *qnrS*	IncY	Plasmid	NZ_LR883966.1
P9‐1‐8‐1	1	1	*bla*_CMY‐2_	—	Chromosome	—
	2	15	*aadA22*, *aph(3′)‐Ia*, *aph(6)‐Id*, *aph(3″)‐Ib*, *sul2*, *sul1*, *aadA2*, *dfrA12*, *aac(3)‐IId*, *bla*_TEM‐1_, *catA2*, *mph*(A), *sul3*, *aadA2*, *tet*(A)	IncY	Plasmid	NZ_CP055093.1
P9‐2‐5‐2	1	13	*sul3*, *aadA2*, *tet*(A), *aadA22*, *aph(3′)‐Ia*, *aph(6)‐Id*, *aph(3″)‐Ib*, *sul2*, *sul1*, *aadA2*, *dfrA12*, *aac(3)‐IId*, *bla*_TEM‐1_	IncY	Plasmid	NZ_CP055093.1
	2	1	*bla*_CMY‐2_	—	Chromosome	—
	3	2	*mph*(A), *catA2*	—	Plasmid	NZ_CP055093.1
P9‐10‐9‐1	1	1	*bla*_CMY_	—	Chromosome	—
	2	14	*catA2*, *mph*(A), *sul3*, *aadA2*, *tet*(A), *aadA22*, *aph(3′)‐Ia*, *aph(6)‐Id*, *aph(3″)‐Ib*, *sul2*, *sul1*, *aadA2*, *dfrA12*, *aac(3)‐IId*	IncY	Plasmid	NZ_CP055093.1
P10‐10‐2‐2	1	8	*bla*_TEM‐1_, *qnrS1*, *aac(3)‐IId*, *dfrA12*, *aadA2*, *cmlA1*, *aadA1*, *sul3*	IncFIB(K)	Plasmid	NZ_CP139126.1
	2	3	*tet*(M), *floR*, *tet*(A)	IncX1	Plasmid	NZ_AP027819.1
P10‐10‐2‐4	1	8	*bla*_TEM‐1_, *qnrS1*, *aac(3)‐IId*, *dfrA12*, *aadA2*, *cmlA1*, *aadA1*, *sul3*	IncFIB(K)	Plasmid	NZ_CP139126.1
	2	3	*tet*(M), *floR*, *tet*(A)	IncX1	Plasmid	NZ_AP027819.1
P10‐10‐10‐1	1	8	*aadA2*, *cmlA1*, *aadA1*, *sul3*, *bla*_TEM‐1_, *qnrS1*, *aac(3)‐IId*, *dfrA12*	IncFIB(K)	Plasmid	NZ_CP139126.1
	2	10	*tet*(A), *aadA5*, *dfrA17*, *floR*, *bla*_TEM‐135_, *aac(3)‐IId*, *sul3*, *aadA1*, *cmlA1*, *aadA2*	IncFII	Plasmid	NZ_CP029058.1/NZ_CP024157.1
	3	3	*tet*(M), *floR*, *tet*(A)	IncX1	Plasmid	NZ_AP027819.1
P10‐10‐11‐6	1	8	*bla*_TEM‐1_, *qnrS1*, *aac(3)‐IId*, *dfrA12*, *aadA2*, *cmlA1*, *aadA1*, *sul3*	IncFIB(K)	Plasmid	NZ_CP139126.1
	2	3	*tet*(M), *tet*(A), *floR*	IncX1	Plasmid	NZ_AP027819.1

^a^ The highest similarity to each plasmid‐derived contig.

**FIGURE 4:**
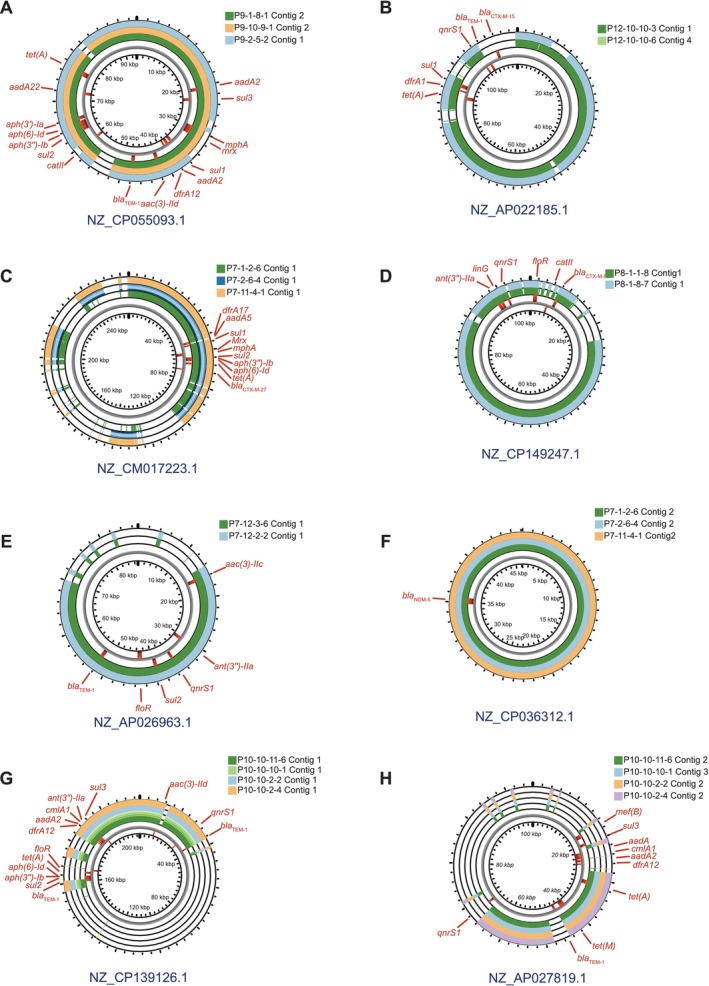
Comparison of plasmid sequences. Sample sequences were compared with homologous plasmid sequences detected from the plasmid databases. These circular maps were drawn by the Proksee website. ARGs locations were drawn by CARD in Proksee.

To understand plasmid structures, homologous plasmid sequences to each plasmid‐derived contigs of the representative *E. coli* isolates were identified by BLAST search among the 22,089 plasmid sequences collected from the RefSeq database. The he highest similar plasmid sequence to each plasmid‐derived contig was designated as the reference plasmid sequence (Table 3). By the BLAST search using plasmid‐derived contigs as a query, it was revealed that the query cover rates against the reference plasmid sequences were between 74% and 100% (Figure 4). Especially, plasmids carrying *bla*_NDM‐5_ exhibited particularly high similarity, with over 200 sequences retrieved from the RefSeq database showing 100% query coverage rate, implying highly conserved plasmid structures.

## 4 Discussion

This study proposed a method that was applicable to AMRB monitoring, to monitor ARGs in AMRB strains isolated from environmental water samples collected. Like the target AMRB species had been determined in advance in conventional research investigations, it would be necessary to determine which AMRB species should be monitored in the target areas or target populations. Both Gram‐positive and ‐negative bacteria would possibly exist as AMRB in the environment and there could be various AMRB species. Although the presence of Gram‐positive bacteria in environmental water cannot be denied, we focused on Gram‐negative bacteria, especially Enterobacterales, in this study by. Because there was potential pathogenicity to humans and it could be important to observe the dynamics of Gram‐negative bacteria when considering their impact on human health. It could be decided whether Gram‐positive or ‐negative AMRB were to be monitored depending on the purpose for example, human health or livestock farming, and the area being monitored.

There are some points to consider when installing the AMRB monitoring system. The first is how to determine the monitoring area. In many cases, there could be many factors to consider when setting up the monitoring area, and these would vary depending on the area. The second is how to determine how frequently and how many cases (samples) to monitor. This is also difficult to determine when the spread of AMRB is unknown in most areas of environmental water. In any case, it is important to conduct as much AMRB monitoring on a pilot scale as possible and collect basic information using a relatively inexpensive method such as the one proposed in this study that could collect genetic information on AMRB.

Our proposed method can be divided into four parts. Namely, (1) sampling and selection of AMRB strains by solid media containing antibiotics, (2) bacterial species identification, (3) phylogenetic classification of AMRB strained for further analysis, and (4) WGS of selected AMRB strains and analysis of data obtained from WGS.

In this study, surface water samples were collected using a seed swab kit, and consequently sufficient AMRB growth was obtained after the enrichment culture. The sampling method is also related to quantitativity and the detection limit of AMRB in AMRB monitoring. Further consideration is needed, including reference to previous studies (Monte et al. 2025; Lima et al. 2025).

To select AMRB present in environmental water two antibiotics, CTX and CIP, were used in this study. CTX is designated by the CLSI as an antibiotic for screening ESBL‐producing bacteria (CLSI 2025). In this study, in addition to ESBL‐producing bacteria, carbapenem‐resistant bacteria that is, *bla*_NDM‐5_‐positive *E. coli* strains, were also selected in CTX‐containing media. Resistance to CTX occurs when bacteria acquire *bla*_CTX‐M_ (Bevan et al. 2017), carbapenemase producing genes including *bla*_KPC_, *bla*_OXA‐48_, *bla*_IMP_, and *bla*_NDM_, and drug efflux pump genes (Jean et al. 2022). CTX‐M type β‐lactamase producing genes, *bla*_CTX‐M_ is classified into several groups based on differences in its sequence, and it is known that there are regional differences in the *bla*_CTX‐M_ groups possessed by ESBL‐producing bacteria (Bevan et al. 2017). Similarly, there are regional differences in carbapenemase producing genes, for example, *bla*_NDM_ is common in South Asia and Southeast Asia, and *bla*_IMP_ is commonly detected in Japan (Jean et al. 2022). Our data suggested that it is possible to detect not only ESBL‐producing bacteria but also various types of carbapenem‐resistant bacteria by using CTX‐containing media.

CIP was also used to select AMRB that are distributing in the environmental water samples. In general, bacteria become resistant to new quinolones through mutations in DNA gyrase genes or topoisomerase genes, or through the acquisition of quinolone resistance genes such as *qnrA*, *qnrB*, and *qnrS (*Khanna et al. 2024; Azargun et al. 2020). The proportion of bacteria resistant to new quinolones is gradually increasing, and AMRB isolated from the environment are often resistant to new quinolones as well. Furthermore, bacteria resistant to new quinolones often also carry other ARGs.

To identify the bacterial species of Gram‐negative rods from the Enterobacteriaceae family, full‐length 16S rRNA was determined by amplicon sequencing using Nanopore sequencer (Komiya et al. 2022). In this study, we hired 16 kinds of barcoded primers to amplify full‐length 16S rRNA for Nanopore sequencing. By our results, the full‐length analysis of 16S rRNA using one Flongle cell enabled simultaneous identification of at least 400 bacterial strains. As shown in Table 2, various AMRB species were detected, suggesting this study's species identification step was effectively done.

It is essential to understand the genetic characteristics of AMRB species detected by AMRB monitoring through WGS. Because AMRB monitoring requires a lot of labour and expense, WGS must be performed as efficiently as possible. The genetic diversity of AMRB that is spreading in the environment is not well investigated, and there is few information on how to select target AMRB strains for WGS. In this study, phylogenetic grouping was performed to select strains for WGS. Phylogenetic grouping methods are well established for some species, such as *E. coli* , but for many species, it has not yet been enough. For instance, not all the genes of the conventional multilocus sequence typing (MLST) scheme are not always amplified in certain AMRB strains especially obtained from the environments (Ajoseh et al. 2025; Tafoukt et al. 2017), and consequently sequence types of these AMRB strains would not be determined.

The WGS performed in this study revealed many points, such as the relative position of the AMRB strain on the phylogenetic tree (Figure 2), the location of carried ARGs that is, on chromosome or on plasmid, and line‐up of ARGs, and the extent to which the AMRB strain had accumulated ARGs (Table 3).

Regarding plasmids carrying ARGs, it was possible that the evolution of plasmids differs depending on ARGs and plasmid replicons detected in the plasmids. To understand the evolution of plasmid carrying ARGs, it is important to observe what kinds of ARGs were carried by plasmids, and how genetic structure of plasmids isolated from many AMRB strains change. Then, comparison of genetic characteristics of plasmids carrying ARGs to others might make it possible to discuss how ARGs accumulate on the plasmids. In the environmental monitoring of AMRB, by creating a database including sample collection time and geographical information and observing the evolution of plasmids, it is highly possible to consider the temporal and geographical distribution of ARG‐carrying plasmids. To achieve this, environmental monitoring of AMRB would need to be further advanced in countries and regions, and the analysed genetic characteristics of AMRB need to be accumulated in the database.

The cost of AMR monitoring method could be reduced by using a Nanopore sequencer. The Nanopore sequencer allowed us to perform high throughput sequencing that could be a major advantage over other analytical methods, such as mass spectrometry and conventional Sanger sequencing by capillary electrophoresis. For single‐gene analyses, such as species identification by 16S rRNA sequencing and phylogenetic classification by *fimH* sequencing, a single Flongle cell were sufficient analysis of more than 400 strains. Consequently, it could reduce the costs and time required for analysis. The Flongle cell (Oxford Nanopore Technologies, FLO‐FLG114) used in this study costs US$ 918.00 for 12 cells, namely, each cell costs US$ 76.5. It means that each strain costs only US$ 0.19 for single gene analysis, which is significantly cheaper than other analytical methods. As for WGS, since 10–20 AMRB strains can be analysed per one Flongle cell, it costs less than US$ 7.65 per each AMRB strain. Therefore, it is suggested that the cost of AMR monitoring proposed in this study can be relatively low and not be a heavy burden for maintaining AMR monitoring.

The last part of discussion is about applicability. In this study, we collected environmental water samples from ponds of the Vietnamese VAC ecosystem. Our study indicated several AMRB species had been distributed in the Vietnamese VAC ecosystem. More importantly, our study could indicate a potential risk that carbapenem‐resistant bacteria were distributed in the ponds attached to VAC ecosystem, implying ponds of the VAC ecosystem worked as reservoirs of AMRB species such as carbapenem‐resistant bacteria. It was suggested that our proposed method was applicable to monitor AMRB in environmental water and that AMRB monitoring in this area using our proposed method could clarify the dynamics of AMRB in the Vietnam's VAC ecosystem.

As above, our results in this study suggested that our proposed method could be applicable to AMRB monitoring system. However, there are at least two major limitations in this study that would be possibly addressed in future. First, quantitative data is essential for monitoring AMRB to understand seasonal fluctuations of AMRB, entire trends of detected AMRB, emerging newly detected AMRB, etc. However, using our proposed monitoring method, only qualitative data would be collected. Depending on contents of the environmental water samples, enrichment culture might be required before selection of AMRB on antibiotic‐containing solid media. Generally, it is not easy to keep composition ratio of bacteria contained in the collected environmental water samples during culturing bacteria, because of growth speed of bacteria would vary among isolates. Antibiotics selection itself could affect the growth speed of AMRB in the collected samples and consequently it became a negative factor in maintaining quantitative data. Even if enrichment culture would be performed, it is important to consider minimising the incubation time or taking supportive information by performing quantitative PCR that would contribute to maintaining the quantitativity.

Second, in this study, to avoid performing WGS on all AMRB isolates, phylogenetic classification was performed to select representative strains for WGS. In case of model species, *E. coli* , *fimH* is available for phylogenetic grouping. However, in many bacterial species, gene(s) that can be used to easily classify genetic lineages, such as the *fimH* in *E. coli* , has not been found. Therefore, for bacterial species other than major AMRB species, such as *Klebsiella* spp., *Pseudomonas aeruginosa* and *Acinetobacter* spp., it is necessary to establish a method that can perform phylogenetic classification like *E. coli* .

## Author Contributions

**Nobuyoshi Yagi:** conceptualization, data curation, formal analysis, funding acquisition, investigation, methodology, writing – original draft. **Itaru Hirai:** conceptualization, data curation, formal analysis, funding acquisition, investigation, methodology, project administration, writing – original draft, writing – review and editing. **Sora Miyazato:** formal analysis, investigation. **Bui Thi Mai Huong:** resources, writing – review and editing, project administration.

## Funding

This work was supported by Japan Society for the Promotion of Science (KAKENHI 23K09672 and KAKENHI 24K20197).

## Conflicts of Interest

The authors declare no conflicts of interest.

## Supporting information


**Table S1:** The summary of isolates information.
**Table S2:** The summary of reference genome sequences used for drawing ORF tree.

## Data Availability

The data that support the findings of this study are available on request from the corresponding author. The data are not publicly available due to privacy or ethical restrictions.
